# Neonatal Ketamine Alters High-Frequency Oscillations and Synaptic Plasticity in the Subiculum But Does not Affect Sleep Macrostructure in Adolescent Rats

**DOI:** 10.3389/fnsys.2020.00026

**Published:** 2020-05-26

**Authors:** Francesca M. Manzella, Srdjan M. Joksimovic, James E. Orfila, Brier R. Fine, Robert M. Dietz, Dayalan Sampath, Hanna K. Fiedler, Vesna Tesic, Navya Atluri, Yogendra H. Raol, Vesna Jevtovic-Todorovic, Paco S. Herson, Slobodan M. Todorovic

**Affiliations:** ^1^Department of Anesthesiology, University of Colorado Anschutz Medical Campus, Aurora, CO, United States; ^2^Neuroscience Graduate Program, University of Colorado Anschutz Medical Campus, Aurora, CO, United States; ^3^Department of Pediatrics, University of Colorado Anschutz Medical Campus, Aurora, CO, United States; ^4^Department of Neuroscience and Experimental Therapeutics, University of Texas A&M, College Station, TX, United States; ^5^Department of Pathology, University of Colorado Anschutz Medical Campus, Aurora, CO, United States; ^6^Department of Anesthesiology, University of Virginia, Charlottesville, VA, United States; ^7^Department of Pediatrics, Division of Neurology, University of Colorado Anschutz Medical Campus, Aurora, CO, United States

**Keywords:** ketamine, sedation, brain development, EEG, long-term potentiation

## Abstract

Exposure to sedative/hypnotic and anesthetic drugs, such as ketamine, during the critical period of synaptogenesis, causes profound neurotoxicity in the developing rodent and primate brains and is associated with poor cognitive outcomes later in life. The subiculum is especially vulnerable to acute neurotoxicity after neonatal exposure to sedative/hypnotic and anesthetic drugs. The subiculum acts as a relay center between the hippocampal complex and various cortical and subcortical brain regions and is also an independent generator of gamma oscillations. Gamma oscillations are vital in neuronal synchronization and play a role in learning and memory during wake and sleep. However, there has been little research examining long-term changes in subicular neurophysiology after neonatal exposure to ketamine. Here we explore the lasting effects of neonatal ketamine exposure on sleep macrostructure as well as subicular neuronal oscillations and synaptic plasticity in rats. During the peak of rodent synaptogenesis at postnatal day 7, rat pups were exposed to either 40 mg/kg of ketamine over 12 h or to volume matched saline vehicle. At weaning age, a subset of rats were implanted with a cortical and subicular electroencephalogram electrode, and at postnatal day 31, we performed *in vivo* experiments that included sleep macrostructure (divided into the wake, non-rapid eye movement, and rapid eye movement sleep) and electroencephalogram power spectra in cortex and subiculum. In a second subset of ketamine exposed animals, we conducted *ex vivo* studies of long-term potentiation (LTP) experiments in adolescent rats. Overall, we found that neonatal exposure to ketamine increased subicular gamma oscillations during non-rapid eye movement sleep but it did not alter sleep macrostructure. Also, we observed a significant decrease in subicular LTP. Gamma oscillations during non-rapid eye movement sleep are implicated in memory formation and consolidation, while LTP serves as a surrogate for learning and memory. Together these results suggest that lasting functional changes in subiculum circuitry may underlie neurocognitive impairments associated with neonatal exposure to anesthetic agents.

## Introduction

Each year, millions of infants and young children are exposed to sedative/hypnotic and anesthetic agents (McGowan and Davis, 2008). These agents are traditionally γ-aminobutyric acid subtype A (GABA_A_) receptor agonists, such as sevoflurane and propofol, and N-methyl-D-aspartate (NMDA) receptor antagonists, such as ketamine. However, exposure to sedative/hypnotic and anesthetic drugs during the neonatal period is associated with profound neurotoxicity and cognitive impairments in rodent and non-human primate models (Jevtovic-Todorovic et al., 2003; Paule et al., 2011; Brambrink et al., 2012; Atluri et al., 2018; Talpos et al., 2019). Currently, there is conflicting evidence on the effect of sedative-hypnotic and anesthetic drugs in infants and young children. Similar to studies conducted in non-human primates, some research suggests that a single, brief exposure may not result in neurodevelopmental delay (Wilder et al., 2009; Pick et al., 2011; McCann et al., 2019). However, there is also some evidence to suggest that repeated exposure to anesthetics during infancy can lead to cognitive impairments in humans (Wilder et al., 2009; Ing et al., 2014). There is also consensus between these studies indicating the need for more research in assessing the risk of future neurocognitive impairment with repeated or prolonged neonatal anesthesia exposures. Because prolonged exposures are less common, they are vastly underrepresented in clinical research (Bartels et al., 2018).

One of the most widely used anesthetics in infants is ketamine, specifically for its effectiveness, availability, and low cost (Bhutta, 2007). It is well established that neonatal exposure to ketamine is associated with developmental neurotoxicity and long-term neurocognitive impairment in rodents and non-human primates, yet there has been little research studying its long-term effects on neuronal physiology, such as its effects on neuronal oscillations when given during infancy (Paule et al., 2011; Brambrink et al., 2012; Atluri et al., 2018; Talpos et al., 2019). Although there is little research on short- or long-term physiological outcomes of neonatal ketamine exposure, its acute effects in adults point to changes in neuronal oscillations, specifically in the high-frequency bands during wake and sleep (Ahnaou et al., 2017; Ye et al., 2018).

Ketamine exposure in different species can create aberrant short-term increases in gamma during wake and sleep periods; although, it is unknown if these effects have a lasting impact (Ahnaou et al., 2017; Richardson et al., 2017; Mahdavi et al., 2019). We speculate that possibly children who receive ketamine either as a one-time anesthetic or as a sedative/hypnotic during long-term hospital stays may also develop these short-term aberrant increases in gamma oscillations. Hence, we propose that changes in high-frequency gamma oscillations during sleep may be an underlying mechanism by which neonatal anesthetics contribute to potentially worrisome cognitive outcomes. However, until recently there has been little research on how exposure to neonatal sedative/hypnotics and anesthetics affect long-term sleep macrostructure, corresponding neuronal oscillations, and synaptic plasticity.

The main purpose of this study is to examine the long-term effects of neonatal ketamine on sleep oscillations and synaptic plasticity in the rat subiculum. The subiculum is especially vulnerable to neurotoxic insult from neonatal anesthesia (Atluri et al., 2018; Joksimovic et al., 2020). It is also a key relay center between hippocampal complex and various cortical and subcortical structures and can independently generate gamma oscillations needed for memory consolidation (O’Mara et al., 2009; Jackson et al., 2011). Thus, neurotoxic damage to the subiculum may contribute to physiological dysfunction contributing to learning impairments. We hypothesized that exposure to ketamine during the peak of synaptogenesis in a rat model at postnatal day 7 may disrupt long-term sleep macrostructure and neuronal oscillations in the subiculum. We also hypothesized subicular dysfunction would manifest as a disruption of synaptic plasticity. We used *in vivo* cortical and subicular electroencephalogram (EEG) and local field potential (LFP) recordings for characterizing sleep macrostructure and neuronal oscillations during sleep, and we used *ex vivo* electrophysiology to measure changes in long-term potentiation (LTP) as a surrogate for memory formation in the subiculum. Together these studies will help expand on understanding the underlying mechanisms by which exposure to neonatal sedative/hypnotics and anesthetics contribute to poor neurocognitive outcomes.

## Materials and Methods

### Animals

All experiments were approved by the Institutional Animal Care and Use Committee at the University of Colorado Anschutz Medical Campus and adhered to the NIH Guide for the Care and Use of Laboratory animals. Ketamine exposure experiments were conducted in Sprague-Dawley (Envigo, US) rat pups of both sexes. Pups were housed with their mothers until initiation of experiment. Animals were maintained on a 14:10 light-dark cycle and had access to food and water *ad libitum*.

### Drugs and Chemicals

Ketamine (Par Pharmaceutical, Woodcliff Lake, NJ, USA) was diluted with saline at a concentration of 20 mg/ml and delivered to rat pups *via* intraperitoneal injection (IP) at a volume of 2 μl per gram body weight for a total dose of 40 mg/kg.

### Ketamine Exposure

**Figure 1:**
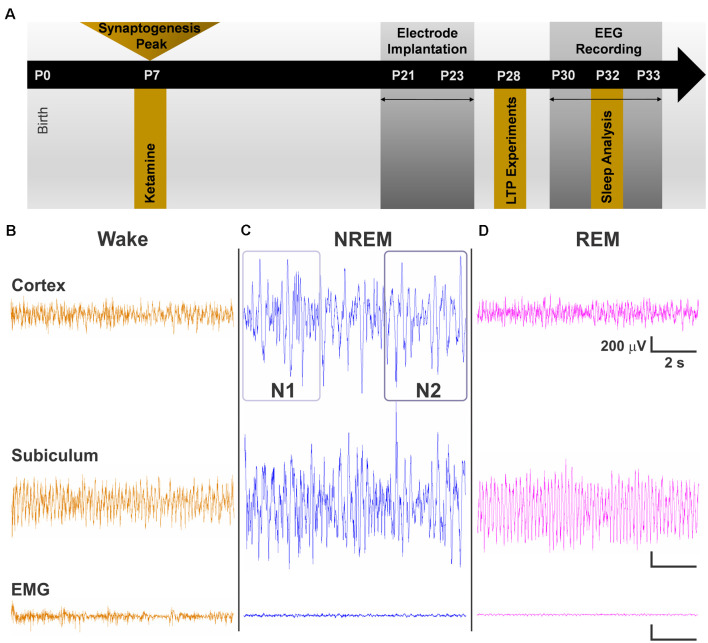
**(A)** Schematic representation of the timeline of events for the experiment. Exposure to a 12-h regimen of ketamine 40 mg/kg occurred at P7 during peak synaptogenesis. A subset of animals was used for long-term potentiation (LTP) experiments at P28. The other subset were weaned from their mothers between P21 and P23. Electroencephalogram (EEG) recordings began at P30 after rats recovered from surgery, and data were obtained from day 3 of recordings at P32. **(B–D)** Representative traces of cortical EEG, subicular local field potential (LFP), and of electromyography (EMG) signals used alongside time-locked videos to score sleep. **(B)** Traces during wake represent an animal that was freely and actively moving as noted by its high-frequency EMG. **(C)** Representative traces during non-rapid eye movement sleep (NREM) show low frequency cortical oscillations and minimal movement in EMG. N1 and N2 were distinguished by differences in the predominance of delta oscillations, with delta comprising >50% epoch in N2. **(D)** Cortical oscillations during REM are similar to those of the Wake stage but with a nearly flat line in the EMG.

At postnatal day (P) 7, rat pups were removed from their home cages and randomly assigned to receive exposure to either the ketamine condition or the saline sham condition. This time period corresponds to the peak of synaptogenesis when animals are most vulnerable to the effects of anesthesia (Jevtovic-Todorovic et al., 2003). Those in the ketamine condition were injected with ketamine 40 mg/kg every 2 h for 12 h (six doses total, Ketamine group). Previous work from our group has found that the sub-anesthetic dose of 40 mg/kg of ketamine given at this regimen is sufficient for inducing hypnosis and apoptosis in a rodent model (Atluri et al., 2018). Pups in the saline condition were only administered saline vehicle at equal volume (Saline group). A heating blanket adjusted to 35°C was used to keep the pups warm during exposure period. Pups appeared pink and healthy and did not show physical signs of hypoxia, such as graying or pallid skin tone. Following the 12-h exposure, Ketamine and Saline pups were allowed to recover and returned to their mothers (Figure 1A, P7 time point).

The dose of 40 mg/kg was a sedative/hypnotic dose in the rat pups. Although all rat pups were not showing visible signs of spontaneous movement and righting reflex, we observed that they still retained the subanesthetic withdrawal reflex upon pinching their leg with a forceps (data not shown). Thus our data show detriments at sub-anesthetic doses. During surgical interventions, children may receive higher doses to induce full anesthetic effect or sedative/hypnotic dose for prolonged sedation, usually in the intensive care unit setting. Hence, our experimental paradigm is more relevant to the later scenario although any relevance of our study to humans would need to be scrutinized in carefully designed clinical studies.

### Electrode Implantation and EEG Acquisition

To study the effects of neonatal hypnosis/anesthesia on sleep, a subset of Ketamine and Saline animals underwent stereotaxic surgeries between P21 and P23 (Figure 1A, P21 time point). Anesthesia was induced at 3% isoflurane *via* inhalation and maintained between 0.5–2%. Lidocaine (1%) was applied at the incision site as a local anesthetic. Because both groups were past the critical period for anesthesia induced neurotoxicity, there was no concern in exposing the Saline group briefly to isoflurane for surgery. A screw electrode was implanted with stereotaxic coordinates in the range of barrel cortex: AP −2.8, ML 3.0. For studying LFPs in the subiculum, animals were also implanted with a depth electrode (AP −4.8, ML 2.4, DV 2.5). Two screw electrodes were implanted just caudal to lambda: one left of the midline, which served as the ground electrode and one right of the midline, which served as the reference electrode. An insulated silver wire hook was inserted in the nuchal muscle to measure electromyography (EMG). Dental acrylic was used to fix the electrodes to the skull, forming the EEG headpiece. Animals were treated post-operatively with Banamine (2.5 mg/ml subcutaneously) for analgesia every 24 h for 48 h.

Animals were housed individually and given at least a week to recover from surgery and to adjust to the headpiece. EEG recording and acquisition took place between P30–33 (Figure 1A, P30 time point). EEG signals were recorded using the Pinnacle system (Pinnacle Technology, Inc., Lawrence, KS, USA) alongside synchronized, time-locked video. We acquired the EEG signals using a 1–500 Hz bandpass filter, which were digitized at 2,000 Hz and stored on a hard disk for offline analysis.

### Sleep Macrostructure and Analysis

EEG and EMG for sleep analysis was taken from day three of recording at P32 so that animals had substantial time to acclimate to the recording device. We separated the recordings into the light and dark cycles (14:10 cycle), and the data were sampled over three continuous hours of the light cycle and three continuous hours of the dark cycle. These were analyzed first separately and then together.

Sleep stages were divided into Wake, non-rapid eye movement sleep (NREM), and rapid-eye movement sleep (REM). Because NREM encompasses a wide range of neuronal oscillations, we further subdivided it into NREM 1 (N1) and NREM 2 (N2). In addition, NREM and REM stages were combined to study effects on overall sleep vs. wake behavior (Sleep). Sleep stages were manually scored in 10 s epochs using Sirenia Sleep Pro (Pinnacle Technology, Inc., Lawrence, KS, USA). Manual sleep scoring was aided by visualization of epoch by epoch spectral plots. Wake stages were characterized by high frequency, mixed amplitude cortical EEG and high frequency EMG when animal was moving (Figure 1B). N1 stages were characterized by low frequency, high amplitude EEG and minimal signal in the EMG (Figure 1C). N2 was differentiated from N1 in that it contained a larger proportion of lower frequency oscillations (1–4 Hz) in at least 50% or more of the epoch (Neckelmann and Ursin, 1993; Wei et al., 2019; Figure 1C). We confirmed the presence of low frequency oscillations by dominance of slow and delta power in the epoch’s power spectral plot. Finally, REM stages were characterized by high frequency, low amplitude EEG resembling awake periods but with flat line signal in the EMG (Figure 1D). REM stages also corresponded to increase in theta power spectra. Time locked videos were used to confirm behavior. All sleep scoring was conducted with experimenter blinded to the treatment condition.

Sleep macrostructure was analyzed using various sleep behavioral outcomes. First, we measured the average sum time spent in each sleep stage. Switching between different sleep stages was measured as the number of transitions between stages. Although, groups may not show changes in overall time spent in each sleep stage, this may be masked by sleep fragmentation. For example, one group may have fewer, but longer sleep episodes while the other group has more, but shorter sleep episodes. To assess changes in sleep fragmentation, we measured the number of episodes animals spent in each sleep stage, as well as the mean duration of each episode (Jang et al., 2010; Leemburg et al., 2010; Lunardi et al., 2019). A sleep episode was defined as a continuous period of time spent in one stage before transitioning into another different stage. Mean episode length was evaluated by summing the total time spent in each episode and dividing that value by the number of episodes.

### Power Analysis

EEG waveform data from cortex and LFP waveform data from subiculum underwent fast Fourier transform using Sirenia Sleep Pro to obtain power spectra values. Data were divided into six frequency bands: delta (1–4 Hz), theta (4–8 Hz), alpha (8–12 Hz), sigma (9–15 Hz), beta (12–30 Hz), low gamma (30–59 Hz), and high gamma (61–100 Hz). Absolute power values were summed for each sleep stage and divided by the number of episodes for that particular stage. Statistical outliers were determined using the Graphpad outlier calculator and were crosschecked to the original EEG to determine if figures were due to normal physiological variation or to EEG artifact. Only outliers that reached statistical significance of *P* < 0.01 and also contained artifacts were removed from analysis.

### Long-Term Potentiation (LTP)

Hippocampal slices were prepared from rats at P28 after neonatal exposure to ketamine (40 mg/kg) or saline treatment. Rats were anesthetized with 3% isoflurane in an O_2_-enriched chamber. Rats were transcardially perfused with ice-cold (2–5°C) oxygenated (95% O_2_/5% CO_2_) artificial cerebral spinal fluid (ACSF) for 2 min prior to decapitation. The composition of ACSF was the following (in mmol/L): 126 NaCl, 2.5 KCl, 25 NaHCO_3_, 1.3 NaH_2_PO_4_, 2.5 CaCl_2_, 1.2 MgCl_2_ and 12 glucose. Horizontal hippocampal slices (300 μm thick) were cut with a Vibratome 1200S (Leica) and transferred to a holding chamber containing ACSF for at least 1 h before recording.

Synaptically evoked field potentials were recorded from hippocampal slices that were placed on a temperature controlled (31 ± 0.5°C) interface chamber perfused with ACSF at a rate of 1.5 ml/min. For subiculum extracellular recordings, a bipolar electrode was placed in stratum oriens at the very end of the CA1 region. Excitatory post-synaptic potentials (fEPSP) were produced by recording in the stratum-radiatum of the distal dendrites of subicular neurons. The fEPSPs were adjusted to 50% of the maximum slope and test pulses were evoked every 20 s. A 20-min stable baseline was established before delivering a theta burst stimulation (TBS) train of four pulses delivered at 100 Hz in 30 ms bursts repeated 10 times with 200 ms interburst intervals. Following TBS, the fEPSP was recorded for 60 min. The averaged 10-min slope from 50 to 60 min after TBS was divided by the average of the 10-min baseline (set to 100%) prior to TBS to determine the amount of potentiation. Analog fEPSPs were amplified (1,000×) and filtered through a pre-amplifier (Model LP511 AC, Grass Instruments) at 1.0 kHz, digitized at 10 kHz and stored on computer for later off-line analysis (Clampfit 10.4, Axon Instruments). The derivative (dV/dT) of the initial fEPSP slope was measured. For time course graphs, normalized fEPSP slope values were averaged and plotted as the percent change from baseline. Two electrophysiologists (RD and JO) independently verified all LTP results in this report.

### Data and Statistical Analysis

Graphpad Prism 8.0 (GraphPad Software Inc., San Diego, CA, USA) was used for all analyses. Two-tailed independent samples *t*-tests were used to compare groups when equal variances were assumed. Unpaired *t*-tests with Welch’s correction were used for groups for which equal variances were not assumed (F test for equal variances, *P* < 0.05). Cohen’s *d* was calculated to measure effect size. Effect size allows for measurement of the strength of the relationship between the means (Sullivan and Feinn, 2012; Lakens, 2013). Data were considered significant at* P* < 0.05 and are presented in text as mean and standard deviation and graphically as mean and standard error of mean.

## Results

**Figure 2:**
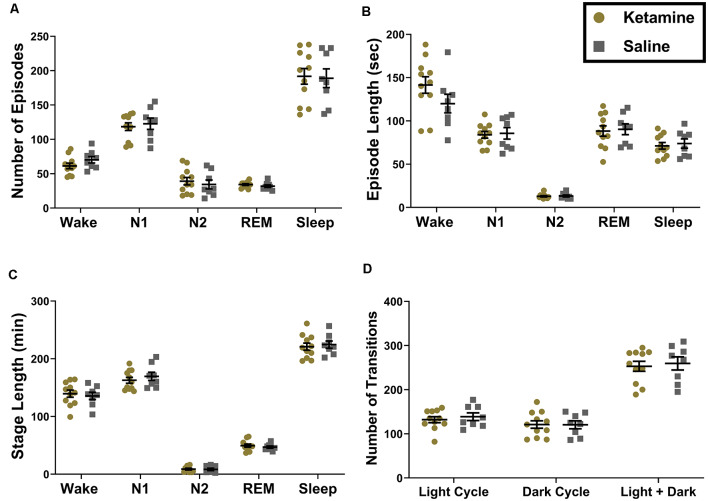
Ketamine exposure during the neonatal period does not result in changes in sleep macrostructure measured across light and dark cycles combined. **(A)** We did not note any changes in the number of episodes in the Wake (*P* = 0.165), N1 (*P* = 0.663), N2 (*P* = 0.581), REM (*P* = 0.322) and total Sleep stages (*P* = 0.880). **(B)** Moreover, the average length of each episode did not differ between groups for Wake (*P* = 0.154), N1 (*P* = 0.823), N2 (*P* = 0.772), REM (*P* = 0.824), and total Sleep (*P* = 0.687). **(C)** The total amount of time spent in each sleep stage also did not change as a result neonatal ketamine exposure across Wake (*P* = 0.685), N1 (*P* = 0.439), N2 (*P* = 0.836), REM (*P* = 0.824), or total Sleep (*P* = 0.691). **(D)** Finally, we measured how often rats transitioned from one sleep stage to another and found no changes in this parameter across the light cycle (*P* = 0.533), dark cycle (*P* = 0.969), and when cycles were combined (*P* = 0.733) in response to neonatal ketamine exposure.

We first measured the effects of neonatal ketamine on different sleep macrostructure parameters in ketamine treated (*N* = 11, six females and five males) and saline treated (*N* = 8, five females and three males) rats. We found that neonatal exposure to ketamine did not result in any significant changes in sleep macrostructure across all of the sleep stages. There were no changes in the number of episodes spent in each stage or the length of each episode (Figures 2A,B). There were also no differences in the total length of each stage (Figure 2C). These results were consistent across the light and the dark cycle; thus, data are presented as combined across cycles. Finally, there were no differences in the number of transitions between stages during the light cycle, dark cycle, or across both combined (Figure 2D).

**Figure 3:**
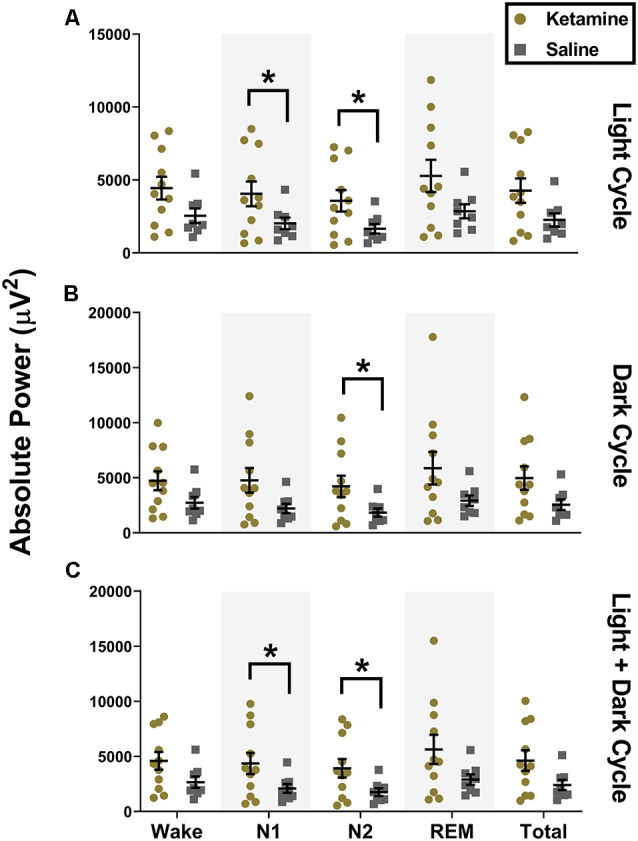
Neonatal ketamine exposure increases subicular low gamma power during N1 and N2.** (A)** Neonatal ketamine exposure resulted in a significant increase in power spectra in the low gamma frequency ranges during N1 and N2 of the light cycle (*P* = 0.049 and *P* = 0.033 for N1 and N2, respectively). However, also during the light cycle, there were no changes in low gamma oscillations during Wake (*P* = 0.078), REM (*P* = 0.065), or across total recording period (*P* = 0.052). **(B)** During the dark cycle, there was an increase in low gamma power during N2 (*P* = 0.040), but not during N1 (*P* = 0.053), Wake (*P* = 0.096), REM (*P* = 0.083), or across total recording period (*P* = 0.057). **(C)** When both cycles were combined, we saw an increase in low gamma during N1 (*P* = 0.049) and N2 (*P* = 0.033). Consistent with results in the light and dark cycle separately, we did not note any changes in low gamma oscillations during Wake (*P* = 0.083), REM (*P* = 0.075), and across the total recording period when both cycles were combined (*P* = 0.052; *P < 0.05).

Although there were no changes in sleep macrostructure, we found that exposure to neonatal ketamine significantly impacted LFP power spectra in the subiculum. During the light cycle, animals exposed to ketamine showed a significant increase in power in the low gamma frequency band during N1 (4,048 ± 2,820 μV^2^, *t*_(13.83)_ = 2.158, *P* = 0.049, Cohen’s *d* = 0.94) and N2 (3,570 ± 2,443 μV^2^, *t*_(13.72)_ = 2.370, *P* = 0.030, Cohen’s *d* = 1.04) compared to saline treated animals (2,025 ± 1,113 μV^2^ and 1,652 ± 947.4 μV^2^ for N1 and N2, respectively, Figure 3A). There were no changes in low gamma oscillations during N1 of the dark cycle. However, the increase in low gamma persisted during N2 of the dark cycle (*t*_(12.81)_ = 2.283, *P* = 0.040, Cohen’s *d* = 0.99). Rats exposed to ketamine (4,214 ± 3,226 μV^2^) had higher low gamma during N2 of the dark cycle than saline controls (1,833 ± 1,065 μV^2^, Figure 3B). When both light and dark cycles were combined, changes were observed again during N1 (*t*_(13.27)_ = 2.170, *P* = 0.049, Cohen’s *d* = 0.95) with ketamine exposed animals (4,360 ± 3,195 μV^2^) having higher low gamma power than saline controls (2,091 ± 1,149 μV^2^, Figure 3C). As with the separated cycles, when data were combined, there were persistent changes in low gamma during N2 (*t*_(13.26)_ = 2.373, *P* = 0.033, Cohen’s *d* = 1.03), ketamine exposed rats (3,919 ± 2,791 μV^2^) had higher low gamma power than saline controls (1,753 ± 1,002 μV^2^, Figure 3C).

**Figure 4:**
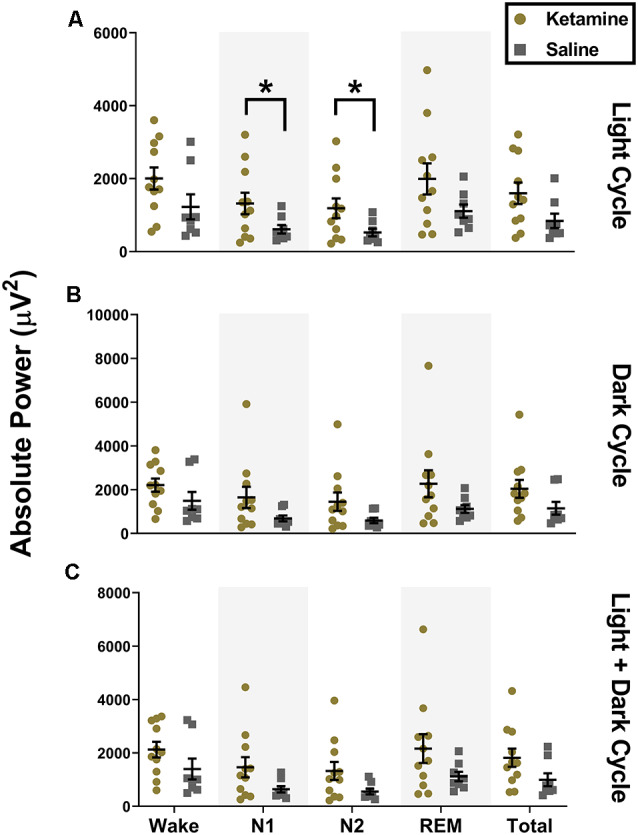
Neonatal ketamine exposure increases subicular high gamma power during N1 and N2 of the light cycle. **(A)** Neonatal ketamine exposure resulted in a significant increase in power spectra in the high gamma frequency range during N1 and N2 of the light cycle (*P* = 0.043 and *P* = 0.040 for N1 and N2, respectively). However, also during the light cycle, there were no changes in high gamma oscillations during Wake (*P* = 0.109), REM (*P* = 0.078), or across total recording period (*P* = 0.066). **(B)** During the dark cycle, there were no changes in high gamma power during N1 (*P* = 0. 083) or N2 (*P* = 0.073). There were also no differences in high gamma oscillations during Wake (*P* = 0.163), REM (*P* = 0.099), or across total recording period (*P* = 0.117). **(C)** When light and dark cycles were combined, changes in high gamma during N1 (*P* = 0.083) and N2 (*P* = 0.051) were not statistically significant. Consistent with results in the light and dark cycle separately, we did not note any changes in high gamma oscillations during Wake (*P* = 0.144), REM (*P* = 0.090), and across the total recording period when both cycles were combined (*P* = 0.087; *P < 0.05).

Similar to what we observed with low gamma during the light cycle, we also saw an increase in high gamma during both N1 (1,319 ± 972.4 μV^2^, *t*_(12.92)_ = 2.241, *P* = 0.043, Cohen’s *d* = 0.97) and N2 (1,187 ± 901.3 μV^2^, *t*_(12.71)_ = 2.282, *P* = 0.040, Cohen’s *d* = 0.99) for ketamine exposed animals compared to saline controls (612.2 ± 327.7 μV^2^ and 523.7 ± 291.9 μV^2^ for N1 and N2, respectively, Figure 4A). However, unlike changes seen with low gamma, statistical changes in high gamma power did not persist into the dark cycle and were not observed when cycles were combined (Figures 4B,C). While there was a significant increase in low and high gamma oscillations in the subiculum, we did not observe changes in gamma oscillations in the simultaneous EEG recordings from the cortex (data not shown).

In addition to analyzing gamma oscillations, we also independently analyzed changes in delta, theta, alpha, sigma, and beta oscillations in the subiculum. However, we did not note any statistical differences between ketamine treated rats and saline treated rats across those frequency bands (data not shown).

**Figure 5:**
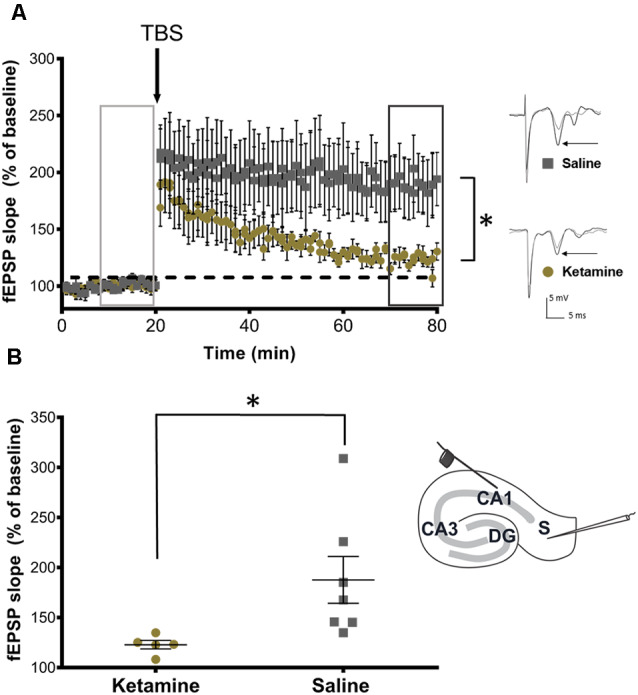
Neonatal ketamine exposure impairs synaptic plasticity in adolescent rats. **(A)** Time plots of fEPSP slope from CA1 to Subiculum pathway in P28 rats exposed to neonatal saline and ketamine. Theta burst stimulation (TBS) arrow indicates the timing of theta-burst stimulation (40 pulses, 100 Hz). Insets indicate sample traces of fEPSPs before (light gray trace) and after (dark gray trace) TBS stimulation in saline and ketamine treated groups. Inset arrows indicate representative trace components used in analyses. **(B)** Quantification of change in fEPSP slope after 60 min following TBS (dark gray box) normalized to baseline (light gray box) in ipsilateral slices and contralateral slices, set at 100%. Rats exposed to neonatal ketamine showed significant impairment in LTP (*P* = 0.032). Each point represents a hippocampal slice (experiment) that was recorded with no more than two experiments per animal used. Inset depicting stimulating electrode placement in stratum oriens at the edge of the CA1 and recording electrode in stratum-radiatum subicular neurons, *P < 0.05.

Finally, to determine the effect of ketamine on hippocampal function in developing brain, extracellular field recordings of subicular neurons were analyzed from P28 rats following neonatal ketamine exposure. Following a stable 20 min baseline, a brief TBS (40 pulse TBS) was applied. The results showed a significant (*P* = 0.032, Cohen’s *d* = 1.47) LTP impairment in ketamine exposed rats (122.80 ± 9.52%, *n* = 5) when compared to saline treated rats [187.60 ± 61.78%, (*n* = 7), Figure 5A]. Figure 5B shows a bar graph summary of these results.

## Discussion

It is well established that treatment with ketamine during the neonatal period of synaptogenesis causes severe apoptosis in rodent and non-human primate models as well as lasting neurocognitive impairments (Ikonomidou et al., 1999; Brambrink et al., 2012; Talpos et al., 2019). We show that in addition to acute neurotoxicity, neonatal ketamine produces significant changes in subicular gamma oscillations during NREM sleep without affecting sleep macrostructure. Moreover, ketamine exposure negatively impacted synaptic plasticity in the subiculum.

During the period of synaptogenesis around postnatal day 7, rodents do not yet exhibit fully developed sleep patterns, as noted by lack of synchronization in the EEG (Gramsbergen, 1976; Khazipov and Luhmann, 2006). An interplay of GABA_A_ and NMDA receptor activation contributes in generating and localizing the spindle burst activity necessary for maturing sleep centers, particularly the thalamo-cortical circuit (Seelke and Blumberg, 2008; Minlebaev et al., 2011). An interplay between GABAergic inputs from the nucleus reticularis thalami and glutamatergic inputs from the ventrobasal thalamus and cortex contribute to the circuit wide burst firing pattern which underlies natural sleep and anesthesia (Steriade, 2006). However, exposure to anesthetic drugs during this period also causes profound apoptosis in both thalamus and cortex (Jevtovic-Todorovic et al., 2003). Despite acute insult to key sleep centers during this critical period, we did not find any effects of neonatal ketamine exposure across any of the sleep macrostructure parameters. These results are in line with results from our group also showing that a 6-h neonatal exposure to 1.5% isoflurane does not result in changes in sleep macrostructure in adolescent rats (unpublished results). However, our results differ from the findings of Lunardi et al. (2019), which found that exposure to a cocktail of midazolam, isoflurane, and nitrous oxide during the neonatal period resulted in increases in REM sleep in adolescent rats (Lunardi et al., 2019). This discrepancy may be due to choice of anesthetic regimen. Because an interplay of GABA_A_ and NMDA activation are required for development of sleep circuitry and subsequent oscillations, treatment with ketamine alone may not be sufficient to elicit a change in sleep macrostructure.

Although we did not note any changes in sleep macrostructure with anesthetic treatment, we did observe changes in neuronal oscillations in the subiculum during NREM sleep. The subiculum receives unidirectional outgoing information from CA1 area, and is thus in a privileged position to serve as a relay center between the hippocampal complex and numerous cortical and subcortical structures (O’Mara et al., 2009). It can also generate low and high gamma oscillations independent of other hippocampal regions, such as CA3 (Jackson et al., 2011). Low and high gamma oscillations generated from various regions of the hippocampal complex are prominent during wake, especially when animals are involved in a cognitive task reviewed in Colgin (2015). They are also present, to a lesser extent, during NREM sleep, where they couple to low frequency oscillations in the 0.1–4 Hz range (Isomura et al., 2006; Le Van Quyen et al., 2010; Valderrama et al., 2012). This coupling is associated with synchronization of neuronal activity and is related to memory formation and consolidation during sleep (Takeuchi et al., 2015). While coupling of gamma oscillations to slow oscillations is necessary for proper cognitive processing, increases in gamma during NREM may not necessarily be beneficial.

Normally, rodents display changes in oscillatory behavior characterized by overall decreases in gamma oscillations while transitioning from wake into NREM sleep in favor of slower range delta oscillations (Corsi-Cabrera et al., 2001). Indeed, we observed these characteristics in our control animals. Instead, an increase in gamma after neonatal ketamine exposure may represent long-term abnormal changes in subicular oscillatory behavior, which may affect accurate transformation of information during sleep resulting in maladaptive plasticity.

During sedation with ketamine in non-human primates, Richardson et al. (2017) found increases in low gamma (around 40 Hz) oscillations and that low gamma amplitude coupled with the phase of slow oscillations under 1 Hz. However, the patterns of gamma coupling to slow oscillations was different in naturally sleeping monkeys, which exhibited high gamma (around 70 Hz coupling to slow oscillations; Richardson et al., 2017). While the authors attribute the differences in coupling to a distinction between natural sleep and sedation, this may also be an abnormal result from ketamine exposure. Indeed, our results support that ketamine has lasting direct effects on hippocampal gamma oscillations during sleep, specifically those generated in the subiculum. Ketamine exposed animals had an increase in gamma power, not seen in saline controls. We observed increases in low gamma oscillations during both N1, which is characterized by sleep spindles and K-complexes, and N2, which is characterized by predominance of slow and delta waves. Moreover, increases in gamma oscillations were more consistent during N2, occurring during both the light and the dark cycle. This indicates a possible relationship between gamma and slower N2 oscillations, which may underlie proper memory consolidation and future cognitive function.

Further evidence of ketamine affecting sleep oscillatory behavior can be observed when mimicking NREM sleep with pentobarbital. Using this paradigm Mahdavi et al. (2019) found that ketamine produced a decrease in cortical and thalamic delta oscillations while transiently increasing gamma, indicating that ketamine alone can increase gamma oscillations independent of sedation (Mahdavi et al., 2019). Although our own results were limited to increases in subicular, and not cortical gamma, it is clear that exposure to ketamine may lead to dysregulation of various gamma generating circuitry and that the subiculum may be particularly vulnerable during development.

In addition to changes occurring directly during sedation and sleep, ketamine can transiently modulate high and low frequency oscillations during wake states. Acute sub-anesthetic doses of ketamine produce an increase in hippocampal low gamma (35–55 Hz) and high frequency gamma oscillations between 120–160 Hz. Acute ketamine exposure also caused increases in broadband gamma EEG between 40 and 140 Hz, which the authors speculated prevented segmenting and coupling of distinct high frequency oscillations to other lower frequency oscillations, such as theta and delta, potentially resulting in behavioral impairments (Ye et al., 2018). Similar to increases in gamma oscillations in the hippocampus, acute ketamine administration also increases gamma power across the cortex and increases cross-frequency coupling between gamma oscillations and lower frequency oscillations in the theta and delta range (Ahnaou et al., 2017). Thus, it is evident that ketamine treatment can increase high frequency oscillations across various brain regions, but especially the hippocampal complex. Although we did not note broadband in the LFPs, an increase in both low and high gamma oscillation may be indicative of lack of segmentation required for proper low frequency coupling during NREM sleep. Moreover, these published effects of ketamine are not long-term and do not persist after a week. Our results indicate long-term changes in neuronal circuitry in animals exposed to neonatal sedative/hypnotics and anesthetics. This may be due to the greater plasticity of the brain during synaptogenesis, in which circuitry is more vulnerable to permanent changes. Specifically, ketamine induced NMDA hypofunction during development may directly play a role on lasting functional and behavioral changes after neonatal exposure.

NMDA hypofunction is closely associated with abnormal elevated gamma band oscillations in patients with schizophrenia. Elevated gamma band oscillations are the result of NMDA hypofunction at the level of parvalbumin GABAergic interneurons, resulting in downstream disinhibition of pyramidal neurons (McNally and McCarley, 2016). Indeed, we found that early exposure to anesthesia leads to lasting hyperexcitability of pyramidal neurons in the rat subiculum, possibly contributing to elevated gamma oscillations (Joksimovic et al., 2020). Furthermore, functional changes associated with schizophrenia can be recapitulated in rodent ketamine models of the disorder, which also display increased gamma oscillations. Increases in gamma oscillations in these models are thought to be associated with underlying sensory and cognitive symptoms of schizophrenia (Ehrlichman et al., 2009; Jadi et al., 2016). Similarly, long-term cognitive impairment is a known outcome of neonatal treatment with anesthetic agents. Neonatal exposure to ketamine results in lasting memory deficits in rodents and non-human primates (Paule et al., 2011; Brambrink et al., 2012; Atluri et al., 2018; Talpos et al., 2019). We propose that long-term changes in gamma oscillations in the subiculum may also play a role in overall learning and memory in animals exposed to ketamine as neonates. As stated earlier, gamma oscillations coupled to low frequency oscillations during NREM sleep play a role synchronizing neuronal activity and facilitating memory formation (Takeuchi et al., 2015).

LTP represents a long-lasting activity-dependent increase in synaptic efficacy and is considered to be the best available model of a cellular mechanism underlying memory formation (Bliss and Collingridge, 1993). This form of synaptic plasticity is most extensively studied in the hippocampal formation, CA3→CA1 (Schaffer collateral) pathway in particular. Much less is known about LTP in CA1→subiculum pathway; however, it appears that synaptic plasticity at this synapse heavily depends on glutamatergic neurotransmission *via* NMDA and AMPA receptors (reviewed in Behr et al., 2009), which may explain its susceptibility to ketamine exposure. It is possible that ketamine-induced NMDA hypofunction during synaptogenesis alters circuitry in the subiculum leading to dysregulation of gamma generation and further decreases in synaptic plasticity, all of which may be detrimental to learning and memory. Our recent data supports this notion since we found that ketamine treated rat pups exhibited decreased learning/memory in adolescence using the radial arm test (Atluri et al., 2018).

Synaptic plasticity and neuronal oscillations are not separate phenomena; on the contrary, the power and relationship of theta and gamma oscillations dictate synaptic plasticity in the hippocampal formation (Dragoi and Buzsáki, 2006). Along these lines, the TBS protocol used in our study was developed to mimic theta-modulated gamma rhythms that occur during animal behavior (Larson and Munkácsy, 2015). Therefore, it could be expected that the overall increase in the power of gamma oscillations may lead to changes in synaptic plasticity in the subiculum, and consequently, affect certain aspects of learning and memory. We found that the LTP induction at the CA1→subiculum synapse was suppressed even in the condition of artificially inducing TBS, which implies that early exposure to ketamine permanently perturbed this particular circuitry.

While we were able to generate an informative understanding of long-term sleep macrostructure after neonatal ketamine exposure, this study was limited in that we did not study changes in sleep microstructure: presence of K-complexes and sleep spindles during N1 and N2. Because spindles are dominant in the sigma frequency band between 9 and 16 Hz, we included sigma in our power analysis (Benington et al., 1994; Seibt et al., 2017; Uygun et al., 2019). However, we did not note any changes between the experimental and control groups (data not shown). Furthermore, we found consistent increases in gamma oscillations consistently during N2, when slow and delta waves are most prominent, but we were unable to assess cross frequency coupling between gamma and delta. Future experiments will benefit from the addition of this assessment, which may further cement that exposure to neonatal ketamine impacts subicular physiology.

In conclusion, our results suggest that neonatal exposure to ketamine produces long-term aberrant increases in gamma during NREM sleep of adolescent rats without affecting sleep macrostructure. These animals also experience a decrease in synaptic plasticity in the subiculum, postulating possible lasting changes in subicular circuitry, which could be contributing to poor neurocognitive outcomes.

## Data Availability Statement

The datasets generated for this study are available on request to the corresponding author.

## Ethics Statement

The animal study was reviewed and approved by Institutional Animal Care and Use Committee at University of Colorado Anschutz Medical Campus.

## Author Contributions

FM, SJ, JO, BF, RD, DS, HF, VT, NA, and YR performed experiments and analyzed the data. FM, SJ, VJ-T, PH, and ST designed the studies, supervised the overall project, and performed final manuscript preparation.

## Conflict of Interest

The authors declare that the research was conducted in the absence of any commercial or financial relationships that could be construed as a potential conflict of interest.
